# A community engagement program to improve awareness for credible online health information

**DOI:** 10.5195/jmla.2024.1899

**Published:** 2024-10-01

**Authors:** Shamly Austin, Emily Hughes, Haiyan Qu

**Affiliations:** 1 saustin@highmarkwholecare.com, Research, Development & Analytics, Highmark Wholecare, Pittsburgh, PA; 2 ehughes@highmarkwholecare.com, Research, Development & Analytics, Highmark Wholecare, Pittsburgh, PA; 3 hyqu@uab.edu, Department of Health Services Administration, University of Alabama at Birmingham, AL

**Keywords:** Health literacy, managed care organization, public-private sector partnership, Medicaid, dually eligible for Medicare and Medicaid, public libraries, program development, MedlinePlus, nutrition program

## Abstract

**Background:**

The volume of online health information available makes it difficult to navigate and check its validity and reliability. A community-based MedlinePlus training program was developed to improve participants’ ability to access credible online health information.

**Case Presentation:**

The program was a public-private partnership between a managed care organization and four local public libraries. A total of eight programs were held between October and November 2017. Each program had a 30-minute cooking demonstration followed by a 30-minute training on access to and navigation of the MedlinePlus website. Program participants were Medicaid beneficiaries, dually eligible for Medicare and Medicaid beneficiaries, and community members from a Pennsylvania county (n=39). A pre-and post-training questionnaire was administered to assess participants’ knowledge and practice, and their ability to access health information on the MedlinePlus website. We conducted a retrospective analysis of the data collected during the MedlinePlus trainings. Results from the Wilcoxon Signed Rank test indicated no statistically significant change in participants’ ability to access information (Z= −1.41, p=0.16) after attending the program.

**Conclusion:**

Although the median pre- to post-program responses improved from ‘incorrect’ to ‘correct,’ the number of programs held, and low attendance might be the reason for non-significant results. Participants reported that the program was informative, the website was comprehensive and user-friendly, and they were impressed by the healthy and inexpensive meal preparation from discount store-bought food. Holding MedlinePlus training programs in conjunction with a cooking program and collaborating with local public libraries might be a promising format that needs additional research.

## BACKGROUND

The internet as a source of information for health has grown. However, the sheer volume of online information available makes it difficult to navigate and verify the reliability and timeliness of information found [1]. The potential harm from inaccurate information could be significant such as disease and medication mismanagement, higher likelihood of adverse health outcomes, and emergency department visits [1]. According to a 2013 Pew survey, about 59% (approx. 185 million) of US adults were online to get insights into their health conditions. In addition, 35% used the internet to diagnose their medical condition or someone else's of which only 53% followed up with a visit to their medical provider [2].

Americans who receive Medicaid or are dually eligible for Medicare and Medicaid beneficiaries have lower income and education, a greater number of chronic conditions, and are more likely to have social needs such as housing, food, and transportation than those who have private health insurance [3, 4]. In accordance with the general population, a 2018 Deloitte survey of US Health Consumers found that 89% of adult Medicaid beneficiaries owned smartphones [5] and nearly 71% of dually eligible beneficiaries residing in communities owned a smartphone or tablet [6], so it is likely that their health information seeking behavior from online resources is also similar to the larger population. Studies have shown that women, younger adults, White individuals, and those with some college degree or advanced degree are more likely to seek online health information [2, 7]. Apart from information on health conditions, individuals also obtain information on drugs, nutrition, and fitness from online resources [2, 7]. Moreover, the seekers of health information are less likely to check the reliability and timeliness of online information. Thus, with the rapid growth in internet use for information on health conditions, it is crucial that vulnerable populations are aware of credible health information sources. Although more research is required to examine the association between exposure to non-credible health information and health behaviors and outcomes [8], acting upon non-credible health information with no follow-up with a health care provider may lead to adverse health outcomes resulting in higher health care utilization and cost.

To improve individuals’ access to credible online health information, we developed a program about the MedlinePlus website to train community members from a southwestern Pennsylvania county. The MedlinePlus website is produced by the National Library of Medicine, the world's largest biomedical library that delivers consumer health information on health, wellness, disorders, tests, drugs and supplements, and genetics in language that health consumers can understand. MedlinePlus offers reliable, up-to-date health information online for free [9]. Previous studies have documented MedlinePlus trainings in a Hispanic community [10], among patients and clinical staff in a community health center [11], and along with a cooking program for community members and other providers in a low-resource setting [12]. However, information on MedlinePlus trainings developed with a public-private partnership that targeted specifically Medicaid and dually eligible beneficiaries in the community is limited. Our objective was to examine program participants’ ability to access online health information after attending a MedlinePlus training program. We hypothesized that there would be an improvement in participants’ ability to access information on the MedlinePlus website after participation in the program.

## CASE PRESENTATION

The program development and implementation were a public-private partnership between a managed care organization (MCO) and four public libraries in a Pennsylvania county. MCOs are health care delivery systems organized to manage cost, utilization, and quality [13]. The MCO manages health care for Medicaid and for the dually eligible for Medicare and Medicaid beneficiaries in Pennsylvania. The reason for selecting the specific county was that the MCO had high beneficiary density, an English-speaking population, and beneficiaries with poor health literacy scores at the census tract level; additionally, beneficiaries had a higher numbers of health conditions, emergency department utilization, and hospitalizations relative to other counties. The MCO partnered with four local public libraries in the county to develop an interactive MedlinePlus training program. The program was developed and implemented during April 2017-July 2018. The target population was adults (22 years or older), Medicaid and dually eligible beneficiaries, and community members. A total of eight programs were scheduled, two programs in each of the four selected libraries. The total number of program participants was 42, of which 39 participants returned the completed questionnaire.

### Participant Recruitment

Recruitment of MCO beneficiaries included approaching those residing within a five-mile radius of each of the four libraries identified using Maptitude mapping software (Caliper Corp.). A separate member list for each library was created with member names and addresses. Three project staff made telephone calls to beneficiaries and informed them about the program. Beneficiaries were offered the MCO's transportation program to and from the venue. Recruitment of community members was through handing out flyers at local grocery stores, exhibition of posters in all four public libraries, library staff handing out fliers to its patrons, and through the social media platform, Facebook.

### The Program

A one-hour program was developed collaboratively by the MCO and local public library directors.

The MedlinePlus training was provided along with an interactive cooking program. The cooking program was a strategy to attract participants to the MedlinePlus training. A local chef demonstrated how to prepare nutritious non-stovetop recipes from local discount stores (Dollar Tree, Dollar General, Family Dollar) purchases (Appendix A). The duration of the cooking program was 30 minutes. Once the cooking demonstration was over, two MCO staff trained the participants on the MedlinePlus website (i.e., how to access the website and search information on health and healthcare issues). The mode of presentation of the MedlinePlus website training was audio-visual. Participants were requested to browse the website using their smart phones. All program participants had their personal smartphones. The MCO staff guided program participants to the MedlinePlus website through the Google search engine. Once landing on the website, the MCO staff walked them through how to search for health topics such as diabetes and hypertension, to access information on drugs and supplements, and to watch videos on health conditions and surgeries. The duration of the MedlinePlus training was 30 minutes including 10–15 minutes of presentation and another 15 minutes for questions and answers and to obtain completed questionnaires. The training was limited to an hour to keep participants’ interest.

### Questionnaire

A questionnaire including both quantitative and qualitative questions was administered to participants that examined their knowledge, practice, and attitudes on online health information seeking and impact of the program (Appendix B). The two-page questionnaire had pre-program questions on one side and post-program questions on the other. The pre-program questions were completed by program participants prior to the beginning of the program and post-program questions after the completion of the program. Responses were measured on a Likert scale. The literacy level of the questionnaire was kept at Flesch-Kincaid grade level of 2.4 to ensure understanding from a health literacy perspective. The questionnaire did not collect protected health information such as name, address, age, gender, and type of health insurance from the participants. The questionnaire was administered between October and November 2017.

### Assessments

Participants’ knowledge was assessed based on whether they had heard about MedlinePlus, which websites they felt gave good facts about health, and how difficult they felt it was to find reliable health information. Participants’ online practices before they accessed medical care was assessed based on three things they would do online before going to medical appointments and how likely they would do these after the training. In addition, participants reported what they learned from the program and were asked to rate it.

The program participants were assessed pre- and post-program on their capability of browsing the MedlinePlus website. The participants were asked to browse MedlinePlus and answer the question of “What is the most prevalent arthritis type in the US?” The answers were measured on a Likert scale as incorrect (scored as 1), partially correct (scored as 2), and correct (scored as 3). Participants who were able to access the website and answer the question correctly were categorized as those with ‘correct’ responses. Participants who were able to access the website, however, got the answer to the question wrong were categorized as those with ‘partially correct’ responses. Participants who got the answer totally wrong were categorized as those with ‘incorrect’ responses. We combined the partially correct and incorrect participant responses into one category as ‘incorrect’ responses. The response categories used for analysis were ‘correct’ and ‘incorrect’. We analyzed the pre- and post-responses using the Wilcoxon Signed Rank test that is widely used to examine pre- and post-measures from related samples [14,15]. In addition, we examined participants’ qualitative responses about the program using content analysis. Quantitative analysis was conducted using STATA version 15 (StataCorp, College Station, TX) and qualitative analysis was conducted with Excel. The Allegheny Singer Research Institute-West Penn Allegheny Health System (ASRI-WPAHS) Institutional Review Board approved the study.

## RESULTS

Of the 39 responses received, 72% (n=28) were from community members and 28%(n=11) were from MCO beneficiaries. Before attending the program, about 15.4% of the participants used the MedlinePlus website for browsing health information, 10.3% had heard about the MedlinePlus but did not use it, and 66.7% had not heard about it. Participants stated that WebMD (59%), MedlinePlus (36%) and NIHSenior Health (26%) provided credible online health information. Other websites used for health information included Dr.Oz, University of Pittsburgh Medical Center, and Mayo Clinic (Table 1).

**Table 1:** Participants’ knowledge, practices, and attitudes around online health information (n=39)

Items	Responses	Percentage
Heard about MedlinePlus	Yes, I have used it.	15.4
	Yes, but not used it	10.3
	No, I do not know about it	66.7
	No response	7.6
Websites that give good facts about health^*^	Medline plus	35.9
	NIHSenior	25.6
	WebMD	59.0
	Wikipedia	20.5
	Everydayhealth	17.9
	All the above	17.9
	None	15.4
	Others	7.7
**Participant practices around online health information**		
Research your health or symptoms online	I never do this	30.8
	I sometimes do this	46.2
	I almost always do this	17.9
	Does not apply to me	5.1
Ask family or friends to help you find health information online	I never do this	51.3
	I sometimes do this	41.0
	I almost always do this	2.6
	Does not apply to me	5.1
Listen to advice from family or friends about how to treat a health problem	I never do this	2.6
	I sometimes do this	20.5
	I almost always do this	61.5
	Does not apply to me	12.8
	No response	2.6
**Hard to find health information online that one trusts**	Very hard	5.1
	A little hard	41.0
	Pretty easy	23.1
	Very easy	5.1
	I do not look online for health information	20.5
	No response	5.2
**After attending the program, participants were likely:**		
Use MedlinePlus to research health questions	Very Unlikely	5.1
	Somewhat Unlikely	7.7
	Not Sure	10.3
	Somewhat Likely	33.3
	Very Likely	38.5
	No response	5.1
Use NIHSeniorHealth to research health questions	Very Unlikely	10.3
	Somewhat Unlikely	7.7
	Not Sure	17.9
	Somewhat Likely	30.8
	Very Likely	25.6
	No response	7.7
Tell others about MedlinePlus	Very Unlikely	5.1
	Somewhat Unlikely	10.3
	Not Sure	5.1
	Somewhat Likely	28.2
	Very Likely	46.2
	No response	5.1
Help others use MedlinePlus	Very Unlikely	5.1
	Somewhat Unlikely	7.7
	Not Sure	10.3
	Somewhat Likely	25.6
	Very Likely	46.2
	No response	5.1
**Program enabled participants to:**		
Find credible online health information you trust	Yes	82.1
	No	2.6
	I am not sure	5.1
	Does not apply to me	5.1
	No response	5.1
Use MedlinePlus to research health condition or treatment	Yes	76.9
	No	2.6
	I am not sure	10.3
	Does not apply to me	5.1
	No response	5.1
Get ready for a health visit	Yes	64.1
	No	2.6
	I am not sure	20.5
	Does not apply to me	5.1
	No response	7.7
Read about a health condition	Yes	76.9
	No	2.6
	I am not sure	10.3
	Does not apply to me	5.1
	No response	5.1
**Participant rating of the program**	Excellent	61.5
	Good	25.6
	Not sure	0
	Not good	0
	Poor	2.6
	No response	10.3

* The total does not add up to 100 as the item was a multiple response.

Only 18% of participants always searched for health conditions before a doctor's visit and 46% did so sometimes. About 41% sometimes asked for help from family and friends to search health information online. About 62% always received advice on health conditions from family and friends and 21% sometimes received advice on health conditions from family and friends. Nearly 46% of participants reported difficulty in finding credible online health information.

After attending the program, about 72% of the participants were very likely or somewhat likely to use MedlinePlus, 74% were very likely or somewhat likely to tell others about the website and 72% reported that they would help others use the website. About 82% reported that the program helped them to search credible online health information. Nearly 77% of program participants responded that the program helped them to access the website to research health conditions. About 64% of participants reported that they would use the website to get ready for a medical appointment, and 21% were not sure about it (Table 1).

Participants' pre-program responses were compared with their post-program responses. On average, the program participants' responses improved from pre-program to post-program. However, a Wilcoxon signed rank test indicated that this improvement was not statistically significant (Z = −1.41, p=0.16) (Table 2).

**Table 2:** Results from Wilcoxon Signed-Rank test examining the change in participants behavior pre- and post-program attendance (n=39)

	Sample Size	Percentile			Wilcoxen Signed-Rank test		
		25^th^	50^th^ (Median)	75^th^	Z	p-value	Effect Size
**Pre-Program**	39	1	1	2	−1.414	0.16	−0.23
**Post-program**	39	1	2	2			

Of the 39 program participants, 90% (n=35) rated the program. About 62%, 26% and 2.6% rated the program as excellent, good, or poor, respectively. Participants’ perceptions about the program were categorized into five themes: very informative, comprehensive, and up-to-date information, user friendly, trustworthy website, and healthy and inexpensive meals. About 62.9% (n=22) of participants provided no response to the open-ended questions. Among the 37.1% (n=13) who responded, 38.4% reported that the program was very informative, 23% thought the MedlinePlus was comprehensive and had up-to-date information, 15.4% reported the website was user friendly, 23% thought the website was trustworthy, and 62% thought the cooking program showed how to prepare healthy and inexpensive meals from discount store food.

Some of the responses to the open-ended question “Write down one or two things you liked learning today that you will use” were as follows.

"Great resource found in one place. It is user friendly. Very informative."

"Website is lot easier to navigate."

"I never thought a healthy meal could be made from products purchased from Dollar store." (Appendix C).

## DISCUSSION

Although our study did not find a statistically significant improvement in participants’ ability to access online health information after attending the program, the public-private partnership, the MedlinePlus training, and the cooking demonstration program from discount store-bought food have implications for future community development programs on improving e-health literacy in under-resourced populations.

Achieving population health objectives all alone is difficult in a rapidly changing and complex world [16]. Partnerships with community-based organizations such as local public libraries to achieve population health objectives is critical as these organizations are trusted entities in the community, have the goodwill of community members, and have wide access to them. Moreover, libraries have evolved from their traditional role of storing, preserving, and issuing tangible books, journals, and CDs to being digital hubs of information exchange and management. In addition, their role now entails empowering communities through knowledge exchange, working with community leaders, local for-profit and non-profit organizations, and patrons to develop programs on early learning initiatives, parent and caregiver education, employment information, health, and social services among others. This new role makes public libraries ideal community partners to collaborate on public health initiatives. Our partnership with libraries relied on clearly specified and shared goals, agreed roles and responsibilities, transparency and accountability, equality of participation, benefits to both parties, and meeting agreed obligations [16, 17].

Furthermore, people seek and share health information from multiple sources such as health care providers, family and friends, health plans, traditional media, social media, pharmaceuticals, and the internet. Studies on online health information seeking behavior show an increasing number of people explored social media platforms for health information [1, 18]. Quality of health information on these platforms is questionable, and social media disseminates both misinformation (conflicts with best scientific evidence) and disinformation (coordinated or deliberate effort to spread misinformation to gain money, power, or reputation) more rapidly and broadly [1, 18]. MedlinePlus is a free, online, comprehensive, health information website for patients, their families and friends [9], access to which they obtain evidence-based information on health and health care. Prior to attending the program, seven in ten had not heard about MedlinePlus or heard about it but did not use it. An equal number of participants after attending the program reported that they intended to use the website for researching health conditions, tell others about the website, and help others use it. Another six in ten reported that they will use the website prior to a medical appointment. The program participants found the website to be informative, comprehensive, up-to-date, user friendly, and trustworthy. Similar positive feedback was documented in previous studies [10–12]. A MedlinePlus training provided along with a cooking program for health care providers and low-income, uninsured, or underinsured women found the website to be valuable and easy-to-use [12]. In another program, librarians and high school peer-tutors conducted MedlinePlus training for their peers, teachers, school administrators, families, and community members in a Hispanic community. The students and teachers reported that the website was very or somewhat useful for their personal and school use [10]. Another program conducted group trainings for clinical staff and one-on-one training for patients in a community health center. Patients suggested they would use MedlinePlus instead of general Google searches or commercially supported online health education sites and after the training a significant proportion of clinical staff recommended the website to their patients [11].

Many of the MCO's Medicaid and dually eligible beneficiaries reside in food deserts or lack financial resources to be on a diet of fresh produce. In addition, discount stores have rapidly grown in low-income neighborhoods, rural areas, and communities of color, and these areas often do not have access to fresh produce [19]. These stores carry non-perishable and processed food. A diet of ultra-processed food leads to greater calorie intake and weight gain than a diet of fresh fruits, vegetables, and other minimally processed foods [19, 20]. Obesity, hypertension, diabetes, and cardiovascular diseases are more prevalent in low-income populations compared with the general US population [21]. Our program introduced a strategy to reduce the prevalence of chronic health conditions in this population through demonstration of cooking healthy recipes from discount store-bought food. The majority of the program participants were impressed by the cooking demonstration.

The study had several limitations. First, the non-significant results may be due to the small number of participants in the study. Although the program was advertised through social media, in-person and in public places, the one-month period of advertisement may not have been enough. Second, the local public libraries were conducting the program for the first time, and it may take time to attract participants; word-of-mouth publicity is considered the best marketing strategy, and it takes time. Third, the program timing (3:30-4:30 p.m., US eastern time) and months (October 19 to November 21, 2017) in which they were conducted may not have been suitable for participation. Fourth, if the program were held a greater number of times in each library, it may have encouraged more participation. Fifth, all program participants had smartphones, however this may not be the case always if the program were to be scaled up. Sixth, the program was held in four public libraries from a single county in southwestern Pennsylvania, hence results are not generalizable.

The objective of the community-based MedlinePlus training was to improve participants’ ability to access credible online health information. With a public-private partnership, we developed and implemented the MedlinePlus trainings along with a cooking program in community settings. The program participants found MedlinePlus to be informative, user friendly, and with the most current and trusted information on health topics. The cooking demonstration was deemed useful as none of the participants knew nutritious meals could be prepared from discount store purchases. Although the program was well received by the participants, statistically favorable results were not achieved, which could be due to fewer people in attendance. Community events have previously shown low participation rates [12, 14] and identifying strategies to improve community participation is key to the success of future similar programs. If the program were to be scaled-up, advertisements about the program would be preferable at least two to three months prior to the scheduled program. Specific community health needs and interests should be identified prior to the development of future programs. Nevertheless, the program provided a unique opportunity to address health-related social needs, specifically health literacy and nutrition among low-income populations. We believe our program can serve as a model for future public-private partnerships on public health initiatives. Partnerships with public libraries are crucial to community health as they are used by a broad segment of the population [22]. Nearly 97% of the US population lives within a public library service area [23] and the libraries have provided a safe place and support for health programs on addiction, nutrition, homelessness, and language services for immigrants, among others [24,25]. The public trust in librarians due to their ability to curate and share reliable information [22,24], their reach [23], and support for health programs [22,24], make them an important ally to collaborate on community health programs. Public libraries in this study played an active role in designing and hosting the program. The library directors were trained on MedlinePlus website so that they can host similar programs in the future and participated in marketing the program to the library patrons through fliers and word of mouth. Also, as part of the program, each of the library received a laptop, projector, and screen to hold the program and for future use. Future program implementation should target strategies on increased participation, and mid-session assessments for facilitators to fix participants navigation issues to increase successful MedlinePlus website use.

## FUNDING DECLARATION

Funding for the study was received from the National Institutes of Medicine/the National Network of Libraries of Medicine Mid-Atlantic Region outreach projects (Award Number: 5UG4LM012342-02).

## Data Availability

The data that support the findings of this study are openly available in Open Science Framework (OSF) at https://osf.io/w9af8.

## SUPPLEMENTAL FILES

**Appendix A:** Recipes

**Appendix B:** Questionnaire

**Appendix C:** Qualitative Responses
