# Supplementary material for: A community engagement program to improve awareness for credible online health information
Source: J Med Libr Assoc. 2024 Oct 7;112(4):341–9. doi: 10.5195/jmla.2024.1899 (PMC11486053; doi:10.5195/jmla.2024.1899)
Supplement: Supplementary file 1 — Appendix A: Recipes [file jmla-112-4-341-s01.docx]

**NON-STOVE TOP COOKING RECIPES**

**Chilled Chicken Primavera**

**Ingredients**

Packets of frozen vegetables-broccoli, carrot, cauliflower (or any vegetables of your choice) —thawed and drained.

1 can cubed chicken, drained

2 tablespoon parmesan cheese

1 tablespoon olive oil

2 teaspoon vinegar

½ teaspoon garlic powder

Pepper and Italian herbs

**Steps**

Toss chicken and vegetables together.

Add rest of ingredients.

Adjust flavor with pepper & herbs.

**Corn, Beans, and Tomato salad**

**Ingredients**

1 can corn, drained and rinsed

1 can green beans, drained and rinsed

1 can black beans, drained and rinsed

1 can diced tomatoes with chilis, drained

½ teaspoon onion powder

1 teaspoon cumin

½ teaspoon pepper

**Steps**

Mix all ingredients in bowl. Let sit an hour, serve.

If you have cilantro add it.

**No bake Oatmeal cookie**

**Ingredients**

1 cup peanut butter

2 cups instant oatmeal

½ cup honey

½ cup raisins

**Steps**

Mix ingredients thoroughly.

Form into 1-inch balls.

Put on cookie sheet and press with fork.

Chill it.

**Ramen Salad**

**Ingredients**

2 packets ramen

1 packet of frozen peas or edamame

2 cans mandarin oranges, drained and rinsed

½ cup unsalted peanuts

½ pound ham diced

2 tablespoon honey

¼ cup olive oil

½ cup vinegar or citrus juice

**Steps**

Mix all together. Let it sit for couple of hours.

Add soy sauce in place of vinegar for an Asian taste/feel.

**Layered Pulled Pork Salad**

**Ingredients**

1 can pulled pork

Chopped romaine hearts

1 can pinto beans rinsed

Cup of salsa

Cup of frozen peas, thawed

¼ cup Fat free ranch dressing

½ teaspoon onion powder

**Steps**

In bowl place chopped romaine.

Top with drained and rinsed pulled pork and onion powder.

Top the pork with pinto beans, then add salsa.

Top with peas and drizzle with ranch dressing.

**Smoothie**

**Ingredients**

1 ½ cup almond milk

1 cup frozen fruit

1/3 can spinach, drained and rinsed

Handful almonds

**Steps**

Place all ingredients in blender and blend until smooth.

**Pumpkin Smoothie**

**Ingredients**

Frozen banana

½ cup of milk or almond milk

2 tablespoon honey

½ cup of yogurt

2/3 cup of pumpkin puree

¼ teaspoon allspice

¼ teaspoon cinnamon

**Steps**

Place all ingredients in blender and blend until smooth.
