# Supplementary material for: A community engagement program to improve awareness for credible online health information
Source: J Med Libr Assoc. 2024 Oct 7;112(4):341–9. doi: 10.5195/jmla.2024.1899 (PMC11486053; doi:10.5195/jmla.2024.1899)
Supplement: Supplementary file 2 — Appendix B: Questionnaire [file jmla-112-4-341-s02.docx]

**APPENDIX B**

**Please fill this side out before the class.**

**Using the Internet to Get Information about Health**

Library Name Date

# Have you heard of MedlinePlus?

- Yes, I have used it. ☐ Yes, but I have not used it. ☐ No, I do not know about it.

# Check off the websites that you think give good facts about health:

- MedlinePlus ☐ NIHSeniorHealth ☐ WebMD ☐ Wikipedia ☐ Everydayhealth
- All the above ☐ None ☐ Other:

# Do you do any of these three things before you go to see a doctor?

(Please check off only one box for each answer)

|  | I never do this | I sometimes do this | I almost always do this | Does not apply to me |
| --- | --- | --- | --- | --- |
| 1. Research your health or symptoms online | **☐** | **☐** | **☐** | **☐** |
| 2. Ask family or friends to help you find health information online | **☐** | **☐** | **☐** | **☐** |
| 3. Listen to advice from family or friends about how to treat a health problem | **☐** | **☐** | **☐** | **☐** |

# How hard is it to find health information online that you trust?

(Please check off only one box)

| Very hard | A little hard | Pretty easy | Very easy | I do not look online for health information. |
| --- | --- | --- | --- | --- |
| **☐** | **☐** | **☐** | **☐** | **☐** |

# Give it a try!

1. Please go to the website in this link: <https://medlineplus.gov/arthritis.html#cat92>.
2. Then try to answer this question:

Which is the most common type of arthritis? (Check off just one answer)

- - Osteoarthritis
  - Rheumatoid Arthritis
  - Gout
  - I do not know

**Please fill this side out after class.**

**How likely are you to do these four things?**

(Please check off only one box for each answer)

|  | Very Unlikely | Somewhat Unlikely | Not Sure | Somewhat Likely | Very Likely |
| --- | --- | --- | --- | --- | --- |
| 1. Use MedlinePlus to research health questions | **☐** | **☐** | **☐** | **☐** | **☐** |
| 2. Use NIHSeniorHealth to research health questions | **☐** | **☐** | **☐** | **☐** | **☐** |
| 3. Tell others about MedlinePlus | **☐** | **☐** | **☐** | **☐** | **☐** |
| 4. Help others use MedlinePlus | **☐** | **☐** | **☐** | **☐** | **☐** |

# Has this class helped you to be able to do these four things?

(Please check off only one box for each answer)

|  | Yes | No | I am not sure | Does not apply to me |
| --- | --- | --- | --- | --- |
| Find health information you trust | **☐** | **☐** | **☐** | **☐** |
| Use MedlinePlus to research your health condition or treatment | **☐** | **☐** | **☐** | **☐** |
| Get ready for a health visit | **☐** | **☐** | **☐** | **☐** |
| Read about a health condition | **☐** | **☐** | **☐** | **☐** |

# Write down one or two things you liked learning about today that you will use.

**Give it a try!**

1. Please go to the website in this link: <https://medlineplus.gov/arthritis.html#cat92>.
2. Then try to answer this question:

Which is the most common type of arthritis? (Check off just one answer)

- - Osteoarthritis
  - Rheumatoid Arthritis
  - Gout
  - I do not know

|  | Excellent | Good | Not Sure | Not good | Poor |
| --- | --- | --- | --- | --- | --- |
| Please check off on box to rate today’s class | **☐** | **☐** | **☐** | **☐** | **☐** |

# Thank you!
