# Supplementary material for: A community engagement program to improve awareness for credible online health information
Source: J Med Libr Assoc. 2024 Oct 7;112(4):341–9. doi: 10.5195/jmla.2024.1899 (PMC11486053; doi:10.5195/jmla.2024.1899)
Supplement: Supplementary file 3 — Appendix C: Qualitative Responses [file jmla-112-4-341-s03.docx]

**Participants’ qualitative responses on the open-ended question “State one or two things you liked learning today that you will use” later**

| **Qualitative Responses** |
| --- |
| Easy economic recipe, great research site |
| Food, Wellness |
| Great resource found in one place. It is user friendly. Very informative. Nice people. |
| I never thought a healthy meal could be made from products purchased from Dollar store |
| It is site that can be trusted |
| Looking up for health information on a website that is up to date and can be trusted.  Learning how to cook healthy, yet inexpensive meals |
| Multiple ways to use the website |
| Program showed that there are possible ways to buy and eat sensibly |
| Reliability of information is so important, Great to know this resource is available |
| The low-calorie meal |
| Very informative |
| The website and food |
| The website is lot easier to navigate |
